# Supplementary material for: Automated design of protein-binding riboswitches for sensing human biomarkers in a cell-free expression system
Source: Nat Commun. 2023 Apr 27;14:2416. doi: 10.1038/s41467-023-38098-0 (PMC10140043; doi:10.1038/s41467-023-38098-0)
Supplement: Supplementary file 1 — Supplementary Information [file 41467_2023_38098_MOESM1_ESM.pdf]

Automated Design of Protein-binding Riboswitches for Sensing Human Biomarkers in a Cell-free Expression System

Authors
Grace E. Vezeau[1], Lipika R. Gadila[2], Howard M. Salis[1,2,3,4*]

[1]Department of Agricultural and Biological Engineering
[2]Department of Chemical Engineering
[3]Department of Biomedical Engineering
[4]Huck Institute Bioinformatics and Genomics Graduate Program, Pennsylvania State University, University Park, PA 16802, USA.
* Corresponding author: Howard M. Salis, salis@psu.edu

Supplementary Figures 1 to 3.

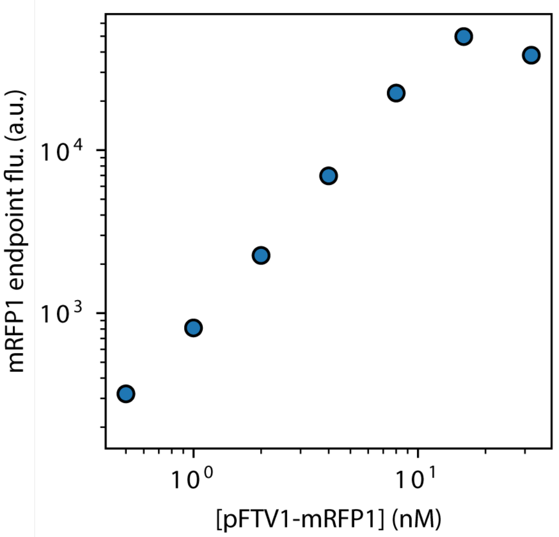

Supplementary Figure 1. mRFP1 fluorescence vs. [pFTV1-mRFP1]. Mean fluorescence levels (blue circles) are proportional to mRFP1-expressing plasmid concentration up to 32 nM. N = 2 biological replicates.

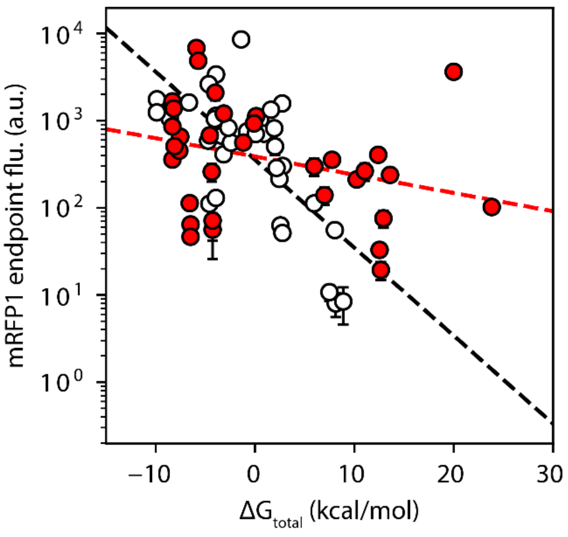

Supplementary Figure 2. mRFP1 fluorescence levels vs. predicted binding free energies controlling translation initiation rates for all 35 riboswitches. The Riboswitch Calculator predicted the binding free energies (ΔG_{total}) of the ribosome-mRNA interactions controlling translation initiation rates for riboswitches in the uninduced and protein-induced states. White circles are mean uninduced mRFP1 endpoint fluorescence levels, while red circles are mean induced mRFP1 fluorescence levels. Dotted lines show the best-fit log-linear line, $\log(mRFP1\ endpoint\ flu.) = \log(K) - \beta\Delta G_{total}$, where the parameter K is a proportionality constant and β is the Boltzmann constant relating free energies to state probabilities. The black dotted line corresponds to uninduced riboswitch fluorescence levels (R^2 = 0.48). The red dotted line corresponds to induced riboswitch fluorescence levels (R^2 = 0.10). The parameter β was previously determined to be 0.45±0.05 in *in vivo* systems across diverse bacterial species (Salis et. al. 2009). In this cell-free, *in vitro* system, we found that β is 0.23, indicating a reduction in the dynamic range of cell-free translation. R^2 = 0.48 for uninduced riboswitches. Error bars represent 95% confidence interval (N = 6 biological replicates for MS2 ON-riboswitches, N = 8 biological replicates for all other riboswitches).

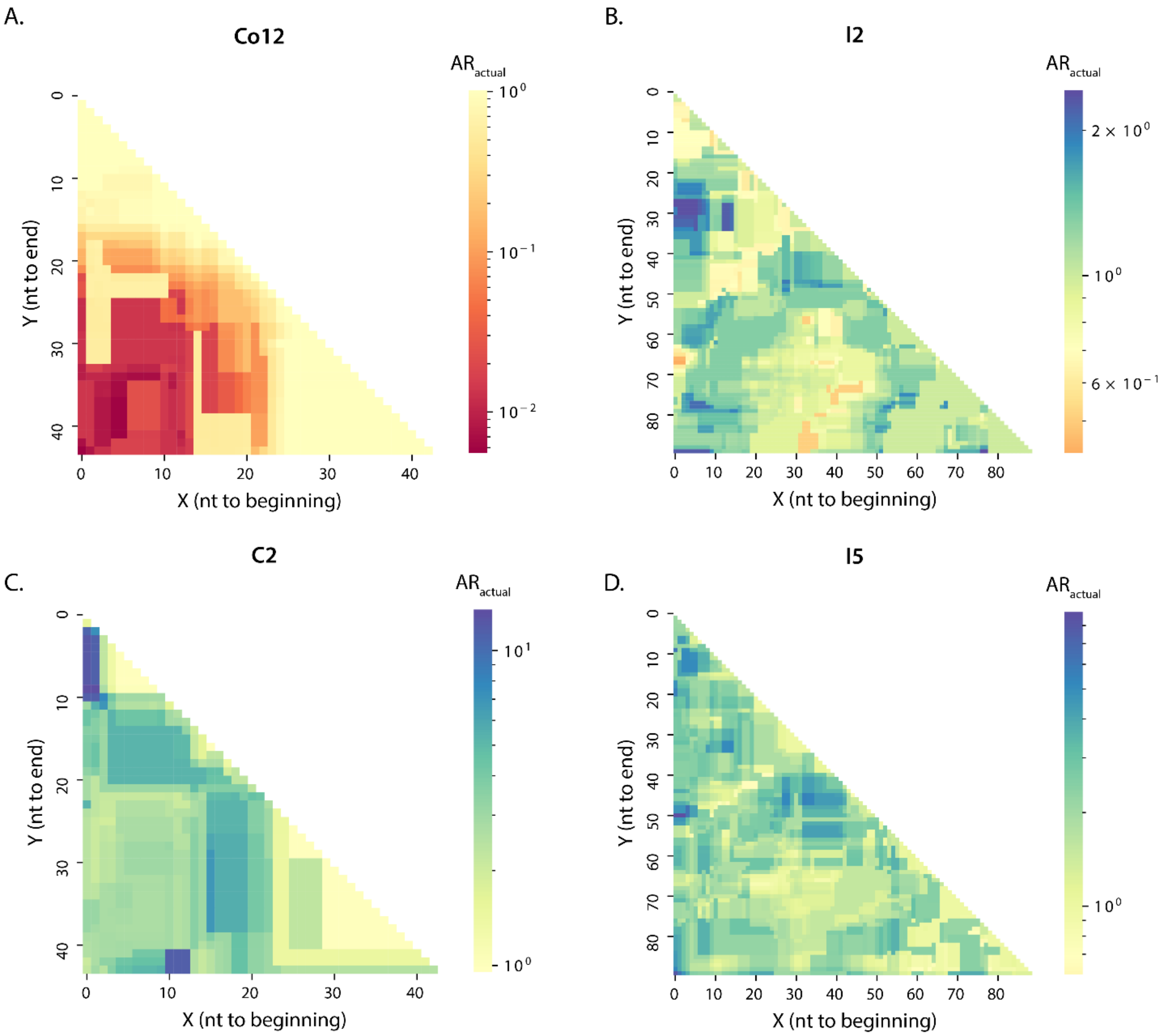

Supplementary Figure 3. The effect of varying the aptamer structural constraint region on mCRP and IL32γ riboswitch predictions. The structural constraint of the aptamer region of riboswitches Co12, C2, I2, and I5 were varied, and the effects on the predicted translation rate ratios (AR_{actual}) were calculated. For all, X represents the distance from the start of the full aptamer sequence to the beginning of the sub-constrained region, and Y represents the distance from the start of the full aptamer sequence to the end of the sub-constrained region. **A.** The effect of varying the size of the mCRP aptamer structural constraint in riboswitch Co12. **B.** The effect of varying the size of the IL32γ aptamer structural constraint in riboswitch I2. **C.** The effect of varying the size of the mCRP aptamer structural constraint in riboswitch C2. **D.** The effect of varying the size of the IL32γ aptamer structural constraint in riboswitch I5.
